# Obstetrics and Gynæcology

**Published:** 1885-05

**Authors:** 


OBSTETRICS AND GYNÆCOLOGY.

*Labor and Child-Bed in Elderly Primiparæ.*—Dr. F. Steinman, of St. Petersburg, contributes to the *Archiv für Gynakologie* ( Band xxii., Heft 3 ) an article on the above subject. His method of investigating it is not the same as that of Dr. Kleinwächter (of whose researches we recently placed an abstract before our readers). Dr. Kleinwächter compared the facts at his disposal relating to elderly primiparæ with those relating to younger subjects in the same clinic, and therefore under circumstances as nearly as possible identical. Dr. Steinman compares the histories of elderly primiparæ at St. Petersburg with averages assumed to be true of labor in general. This method seems to us more open to objection than that of Kleinwächter; for exceptional features shown to be present in the St. Petersburg elderly primiparæ might be partly, at least, due to peculiarities in the patients, or in the working of that particular institution. Kleinwächter, by comparing patients of the same place and under the same conditions, differing only in age, seems to us to have worked on a method more likely to give correct results. With this explanation we put Steinman's conclusions before our readers. It will be seen that in some important respects they agree with Kleinwächter. (1) The only results that are comparable are those of authors who have collected their material in lying-in hospitals; figures based on private practice, out-door maternities, and published cases show exceptionally unfavorable results in elderly primiparæ; an exceptionally high percentage of mal-presentations and narrow pelves, and a correspondingly great frequency of operations, of morbility, and mortality, maternal and infantile. (2) The proportion of elderly primiparæ is greater at St. Petersburg than at Marburg or Munich. (This, we think, illustrates our criticism.) (3) Twins are slightly more frequent than in other cases. (4) Transverse and pelvic presentation are somewhat more frequent than common. Face presentations are rather less common, this being the contrary to what other statistics show. (5) Narrow pelves are more common than in other cases. (6) operations are more frequent, especially the forceps, than in other cases, even with normal pelves; from which it would appear that weak uterine action is common. (7) Spasmodic contraction of the uterus and eclampsia are observed considerably more often than in other cases. (8) With increasing age of the primiparæ, rupture of the perineum is more frequent, and more seldom heals. (9) The morbility is increased correspondingly to the increased frequency of complications shown in the foregoing; and elderly primiparæ are more liable to accidental intercurrent disease during childbed than others. (10) Consequently both the general and the purely puerperal mortality in them is increased. (11) Albuminuria and kidney diseases are seen more often in elderly primiparæ than in others, both in pregnancy and in lying-in. (12) The mortality of children of elderly primiparæ is nearly three times as great as that of others in the St. Petersburg clinic; this increase being mainly due to the large number of children dying during delivery.

*Remote Puerperal Hemorrhage.*—At a recent meeting of the New York Obstetrical Society (*New York Medical Journal*),

Dr. Gaillard Thomas referred to some examples he had recently met with of the condition which the late Dr. McClintock termed "remote or delayed puerperal hemorrhage," in which the bleeding comes on three weeks or a month after labor, when the practitioner has ceased making his visits. The uterus may have contracted after labor, and everything have gone on properly until the ninth day or so, when the physician ceases his daily visits, but from that time the woman begins to lose blood steadily. If she makes a little additional effort, or if anything occurs to cause considerable mental excitement, an exceedingly dangerous hemorrhage may take place, which will require to be checked by the tampon. If sudden and profuse hemorrhage does not occur, requiring the services of a physician immediately, a steady loss of blood in moderate amount may continue for a week or ten days, until the woman becomes very much exhausted. Dr. Thomas has met with many of these cases, and he detailed the particulars of one which were of considerable interest. The lady who formed the subject of the case had ten months before come under his notice for aggravated vaginismus, which quite precluded connection; but after an operation had been performed she immediately conceived. About the end of the seventh month the veins leading from each labium became greatly enlarged, the parts presenting the appearance of a mass of earth-worms of the size of a fist—a condition which Dr. Thomas has seen in so marked a degree only once or twice before. On the ninth day after delivery at the full time hemorrhage came on, and the various remedies, except the tampon, were tried in vain to arrest it; and about three weeks after delivery she was seized with violent and very profuse hemorrhage after she had got out of bed, which reduced her very much. Each time that this occurred it was preceded by the passage of a large blood-clot. Dr. Thomas was now sent for and came prepared for the removal of the remains of membranes, the retention of which he felt sure was the cause of the hemorrhage. Her attendant, however, felt very positive that none of the membranes had been left, as he had examined the placenta very carefully and found no interruption of its continuity existing. Dr. Thomas, feeling equally positive that some of the placenta was still in the uterus, the patient was placed on a table for examination and ether attempted to be employed. At this she sprang up in a state of wild excitement, refusing the inhalation from the recollection of having suffered so much from it during the operation for vaginismus. All persuasion was futile, and Dr. Thomas refused to resort to compulsion, as suggested by her friends, having seen under such circumstances violent mania developed. "I think," Dr. Thomas says, "we cannot be too careful as to doing that which is strongly opposed to the will of a puerperal woman. I would rather run the risk of a violent hemorrhage in this case than have forced the patient to take ether. She was spoken to kindly and put back into bed." Next day she submitted to be etherized, and the canal having been dilated, the curette was passed in and three pieces of placenta, each as large as the last phalanx of an index finger, were removed, very little hemorrhage ensuing. "With regard to the statement so often made, that the placenta has been carefully examined and found entire, it usually amounts to nothing. In the first place, we know that the physician commonly looks at the after-birth hastily, and in a careless manner. Besides, I believe that little pieces may be broken off, and remain behind, which no man could recognize from an examination of the placenta, though he examined it with the utmost care. As in this case, so in all others of delayed puerperal hemorrhage that I have met with, it has been due to retained placenta or membranes. Dr. McClintock mentioned a case in his practice, which, I believe, proved fatal. I have met with some which very nearly proved fatal, and doubtless some of those present have encountered similar cases."

*Laparo-Myotomy.*—A recent number of the *Zeitschrift für Geburtshülfe und Gynäkologie* (Band x., Heft 1) contains an interesting article on the above subject by Dr. R. Kaltenbach, of Giessen. He relates seven cases of the operation in question, and then discusses the general subject. Among his cases, the one that seems to us of most special interest was that of a patient who was able to tell in which ovary ovulation was going on by the presence of burning pain, which began eight days before each menstrual flow, and was felt in the situation of the ovary that was active. The operation was performed three days before the expected advent of the flow. Before it was done the patient indicated the left ovary as the one which was ovulating, and there was tenderness on pressure over its situation. At the operation a freshly-ruptured Graafian follicle was found in this ovary. Dr. Kaltenbach has published in a former paper three other cases; these, with those now published, make ten, of which nine have been successful. Two of them were cases in which a pedunculated fibroid was removed; the remainder cases of supra-vaginal hysterectomy. The fatal case was one of the latter. The pedicle in the former cases was treated by the intra-peritoneal, in the latter by the extra-peritoneal method. The author discusses the different methods of dealing with the stump. Although the extra-peritoneal method has hitherto been attended with the better results, yet Dr. Kaltenbach does not regard its superiority as certain to be permanent. He expects improved results from the mode of intra-peritoneal treatment, which consists in the suture of the peritoneum over the stump, with the ligature of the uterine and ovarian arteries on each side. If the extra-peritoneal operation is done, Dr. Kaltenbach prefers the treatment of the stump after the method of Hager, viz., by chloride of zinc; 100 per cent. solution of which is applied to the surface of the stump, 5 to 10 per cent. solution to the lateral parts of it, and chloride of zinc wool of 2 to 5 per cent. strength packed around the stump.

*The Anatomy and Pathology of the Labia Minora.*—Dr. Henri Carrard, of Bern, contributes to the *Zeitschrift für Geburtshülfe und Gynäkologie* (Band x., Heft 1) a paper on the above subject. He had occasion to examine the labia minora removed on account of hypertrophy; and in order to be able to judge how far these were morbid, he examined a series of healthy labia. He remarks on the scanty information on the subject to be found in literature. Hildebrandt refers *pruritus vulvæ* to a dilatation of the vessels of the nymphæ, basing this view on one case in which these parts looked redder than normal. In Dr. Carrard's case there was pruritus, but he found no evidence of enlargement of the vessels. There is divergence of opinion as to the covering of the labia minora; some say it is skin, others mucous membrane. Dr. Carrard says that although moist, and resembling mucous membrane, it has the histological characters of skin—that it presents papilæ, sebaceous glands and epidermis, while mucous glands are quite absent. There is a similar divergence as to the mode of termination of the nerves which supply the lesser labia. Dr. Carrard finds in their papillæ regularly well-developed Meissner tactile corpuscles, bodies which have hitherto been found only in the palms of the hands and soles of the feet, the nipple, free borders of the eyelid, the mucous surface of the lips, the front of the forearm, and the clitoris. He did not discover spheroidal end bulbs, nor the so-called "genital corpuscles." In the cases with hypertrophy with pruritus he found increase in the connective tissue and in the nerve structures. The nerve structures consisted of Meissner's corpuscles, spheroidal and bulbs (as in the conjunctivæ), and other peculiar bodies which he believes have not been described, although they are very much like the "genital corpuscles" of Krause, and which are something between the two modes of nerve termination just mentioned. Lastly, he has found in the hypertrophied labium adenoid tissue disposed under the surface, mostly around the orifices of the sebaceous glands. He confesses himself entirely in the dark as to the significance of this. The paper is well illustrated.

*The Removal of Clots in Uterine Hæmorrhage.*—All who heard the late Professor Meigs lecture upon *post-mortem* hæmorrhage, were struck by the earnest force with which he exclaimed, "Turn out the clot!" That this rule is not absolute was shown by a case recently cited in a paper read by Dr. Hibbert before the Indiana Medical Society. The patient, fifteen minutes after the birth of her second child, had hæmorrhage. The placenta was largely adherent, and was detached with difficulty, the uterus firmly contracting under the influence of external manipulation and the administration of a full dose of ergot, with a medium dose of morphia and a little alcohol. In a little time the patient fainted, but soon rallied without any attempt at restoring her. The fainting recurred, and spontaneous revival again took place, followed by perfect restoration and rapid convalesence. Dr. Hibbert observes that in this case he purposely availed himself of the remedial agency of syncope, under the belief that the manipulation necessary for the removal of the placenta had caused an oozing from the abraded surface, or had broken a vessel which the uterine contraction had not closed. His hope was that the syncope would favor the formation of a clot that the reëstablishment of the circulation would not remove. He believes that if he had removed this clot, and endeavored to secure a better contraction of the uterus by internal manipulation, by ice or styptics, the patient would have died.—*Philadelphia Medical News*.

*Cramp of the Levator Ani*—Dr. K. Henrichsen, of Odessa, contributes to the *Archiv für Gynäkologie* (Band xxiii., Heft. 1) a short paper on the above subject. Spasmodic contraction of the levator ani muscle has been mentioned by Scanzoni, and Hildebrandt described a case in which the penis during coitus was so grasped by the contraction of the levator ani, that it could not be withdrawn from the vagina while the cramp of the muscle lasted. Contraction of the levator ani constricting the vagina has been described by Marion Sims, Beigel, and we believe Budin. Dr. Henrichsen now describes a case in which digital examination per vaginam excited such spasmodic contraction that the neck of the uterus could not be reached with the finger, owing to the contraction of the vagina. The same spasmodic contraction could be perceived on examination per rectum. The muscle constricting the vagina is presumed to be the levator ani. On reëxamination the following day, none of this contraction was present, and the cervix uteri could be explored with ease. The cramp, while it lasted, was painful to the patient. Dr. Henrichsen, from his experience of this case, is prepared to accept Hildebrant's account of the "penis captivus." As to this, we would ask a question which may be thought coarse, but which we think very pertinent: Given an active man, with his penis well greased, would it be possible for an enemy to hold him captive by grasping that organ? We think not, and therefore cannot help feeling doubtful as to occurrence of incarceration of the penis in any literal sense of the word. But the occurrence of constriction of the vagina we of course do not question.

*Gynæcological Teaching on the Phantom.*—Professor Winckel, of Munich, in a lecture published in the *Archiv für Gynäkologie* (Band xxiii., Heft 2) remarks on the importance to practitioners of some practical training in the performance of the common operations of gynecological surgery, and on the obstacles which prevent the dead subject from being used for the purpose of teaching these manipulations. Winckel has devised a means of giving the requisite demonstrations. He takes an ordinary phantom, such as is used in midwifery lectures, *i. e.*, a pelvis, with limbs, etc. From a female cadaver he cuts out in one mass bladder, rectum, uterus, vagina, and vulva. These structures are thoroughly washed, and soaked for some weeks in corrosive sublimate solution, with a quarter of its volume of glycerine added to destroy putrefactive germs. This remove smells and renders the parts supple. Then with india rubber bands and sutures the parts are fixed in their proper position inside the pelvis of the phantom. When this has been properly done, the teacher can demonstrate on the parts so prepared the use of the different specula; the sound, perineal, vaginal and vesico-vagineal plastic operation; removal of urethral caruncle; incision of cervix uteri; Emmett's operation, etc., in a very satisfactory manner.

*Tissue Change in the Fœtus.*—Dr. Wiener, of Breslau, contributes to the *Archiv für Gynäkologie* (Band xxiii., Heft. 2) a long argumentative article on the question of tissue change in the fœtus. Some regard the changes which take place in the growing fœtus as being almost entirely those of growth, new material being added and assimilated, but little if any cast off and excreted. We cannot in the space at our disposal attempt a summary of Wiener's arguments, which are very exhaustive and detailed, but we quote the conclusion at which he arrives. "The amount of tissue change in the fœtus in utero, although less than that in the infant after birth, is by no means inconsiderable, and probably bears a certain relation to the size and rate of growth of the fœtus; and the excretory products of fœtal tissue change are so abundant that a regular and free secretion of the fœtal kidneys is the consequence."—*Medical Times and Gazette.*

